# Research on Mask-Wearing Detection Algorithm Based on Improved YOLOv5

**DOI:** 10.3390/s22134933

**Published:** 2022-06-29

**Authors:** Shuyi Guo, Lulu Li, Tianyou Guo, Yunyu Cao, Yinlei Li

**Affiliations:** School of Mechanical Engineering, North China University of Water Resources and Electric Power, No. 36 Beihuan Road, Zhengzhou 450045, China; guoshuyi@ncwu.edu.cn (S.G.); 201922126@stu.ncwu.edu.cn (T.G.); z20201040489@stu.ncwu.edu.cn (Y.C.); liyinlei@stu.ncwu.edu.cn (Y.L.)

**Keywords:** object detection, YOLOv5, Coordinate Attention, BiFPN

## Abstract

COVID-19 is highly contagious, and proper wearing of a mask can hinder the spread of the virus. However, complex factors in natural scenes, including occlusion, dense, and small-scale targets, frequently lead to target misdetection and missed detection. To address these issues, this paper proposes a YOLOv5-based mask-wearing detection algorithm, YOLOv5-CBD. Firstly, the Coordinate Attention mechanism is introduced into the feature fusion process to stress critical features and decrease the impact of redundant features after feature fusion. Then, the original feature pyramid network module in the feature fusion module was replaced with a weighted bidirectional feature pyramid network to achieve efficient bidirectional cross-scale connectivity and weighted feature fusion. Finally, we combined Distance Intersection over Union with Non-Maximum Suppression to improve the missed detection of overlapping targets. Experiments show that the average detection accuracy of the YOLOv5-CBD model is 96.7%—an improvement of 2.1% compared to the baseline model (YOLOv5).

## 1. Introduction

COVID-19 is highly contagious and spreads primarily through respiratory and contact transmission. Scientists proved that wearing face masks works on impeding COVID-19 transmission. Consequently, intelligent monitoring of mask-wearing can be accomplished by deploying relevant devices in places with high population flow rates, such as airports and supermarkets, which can effectively improve the efficiency of COVID-19 pandemic prevention and control. Because current mask wear detection devices are susceptible to the effects of a complex environment, such as a surge increase in the number of people to be inspected in a short time, people blocking each other, and other false detections, it is necessary to improve the accuracy and speed of mask wear detection in complex scenarios.

With the development of deep learning tools, target detection models are very effective across different application domains such as vehicle detection [1,2], traffic sign detection [3], face detection [4,5], medical image analysis [6], and target tracking [7,8]. Deep learning-based target identification algorithms include one-stage target detection techniques based on regression analysis and two-stage target detection algorithms based on candidate boxes, as well as two-stage target object detection methods such as R-CNN [9], SPPnet [10], Fast R-CNN [11], Faster R-CNN [12], Mask R-CNN [13], and FPN [14]. The advantage of the two-stage algorithm is that target detection is done in different size regions using the anchor box mechanism, which improves localization and detection precision even more. At the same time, the two-stage algorithm will also generate many candidate boxes, which increases the computing time and the accuracy and speed of detection. The one-stage object detection methods such as YOLO [15,16,17,18], SSD [19], DSSD [20], RetinaNet [21], and EfficientDet [22] obtain target location and classification results through forward inference from the network. This method is much faster than the two-stage detection algorithm. However, the accuracy of localization and detection is slightly lower, and the effect of detecting small targets is less effective.

The remainder of this paper is organized as follows: Section 2 describes related work, Section 3 describes the YOLOv5 network model, and Section 4 describes our improved approaches. Section 5 presents detailed experimental results and discussions, including a description of datasets and related assessment metrics. Finally, Section 6 contains the conclusions.

## 2. Related Work

Existing published work in this domain of face mask recognition consists of two main areas: (1) face mask classification and (2) face mask detection. In ref. [23], the author used the YOLOv3 algorithm for mask-wearing detection, introduced an improved spatial pyramid pooling structure in the YOLOv3 algorithm, and selected Complete Intersection over Union (CIoU) as the loss function; the proposed method achieved 90.2% accuracy. In Ref. [24], to solve the problem of low detection accuracy of wearing masks in dim light conditions, a technique of mask-wearing detection combining attention mechanism with the YOLOv5 network model is proposed, which can reach 92% accuracy under dark lighting conditions. In Ref. [25], the author proposed a novel face mask detection framework named FMD-Yolo. The author employed Im-Res2Net-101 as the feature extractor, which combines the Res2Net module and deep residual network, and applied an enhanced path aggregation network En-PAN for feature fusion to enhance the model’s robustness and generalization ability. In Ref. [26], the author proposed a model consisting of a ResNet-50-based deep transfer learning module and a YOLOv2 mask-detection module and used mean Intersection over Union (IoU) to estimate the best number of anchor boxes. Experimental results show that the average precision using an Adam optimizer as a detector reaches 81%. In Ref. [27], the authors migrated YOLOv3 to the face detection area. They made some improvements to adjust it to the face detection problem, including changing the detection layer and choosing the SoftMax as the loss function. In Ref. [28], the authors presented a system for detecting the presence or absence of mandatory medical masks in the operating room. The overall objective is to minimize the false positive face detections possible without missing mask detections to trigger alarms only for medical staff who do not wear a surgical mask. It can be concluded from the literature that the problems of the current target algorithms can be attributed to face occlusion, variable face proportions, uneven illumination, and density, which seriously affect the algorithm’s performance.

Based on the analysis above, this paper proposes an improved detection model, YOLOv5-CBD, to address the problems of low accuracy and high rate of missed detection caused by occlusion, density, and small-scale mask-wearing detection in public locations. The YOLOv5-CBD model has been improved in three ways. (1) The Coordinate Attention (CA) [29] mechanism is introduced into the feature fusion process to stress critical features. (2) Using the Bidirectional Feature Pyramid Network (BiFPN) to replace the original feature extraction network, which fully integrates high-level semantic and low-level location information and combines features at different scales to detect the small objects challenging to detect. (3) Replacing Non-Maximum Suppression (NMS) with Distance Intersection over Union (DIoU) [30] NMS to improve the identification of overlapping targets.

## 3. The Model Structure of the YOLOv5 Network

**Figure 1:**
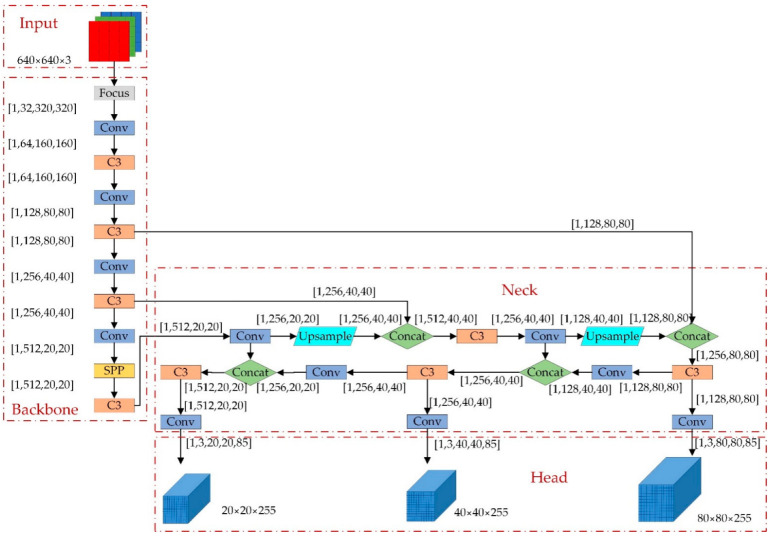
The structure of YOLOv5s.

After several years of update iterations, in 2020, the YOLO series has evolved to YOLOv5, which has improved accuracy and speed over the traditional detection method and has strong real-time performance. Therefore, in this paper, YOLOv5 is selected as the baseline experimental model and used as the baseline for model optimization. The YOLOv5 model consists of four parts: the input layer, backbone network, neck network, and output detection layer. Taking YOLOv5s as an example, the structure is shown in Figure 1.

### 3.1. Input

**Figure 2:**
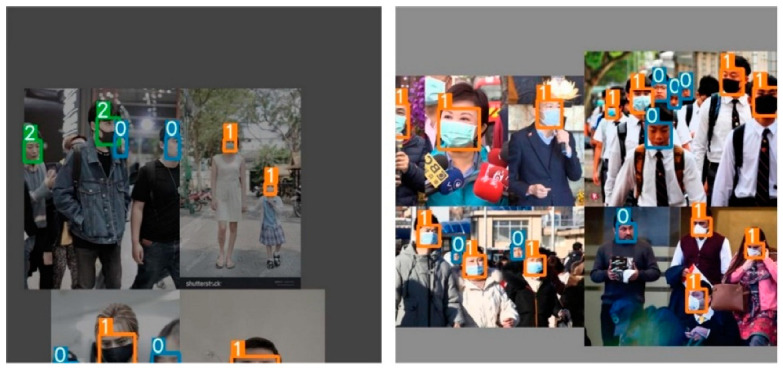
The effect of Mosaic data augmentation.

The input adopts Mosaic data augmentation, adaptive anchor, and adaptive image scaling. Mosaic is a data enhancement method that stitches four randomly picked photos together using random scaling, clipping, and arranging to enrich the data and increase the network’s detection capacity for small objects. Figure 2 is a schematic diagram of the mosaic data enhancement. YOLOv5 adaptively generates various prediction boxes based on the original anchor box during model training and picks the prediction box closest to the genuine box using NMS. Because of the nonuniform size, the adaptive zooming image is scaled to a suitable standard size before being input into the network for detection, avoiding problems such as a mismatch between the feature tensor and the fully connected layer.

### 3.2. Backbone

**Figure 3:**
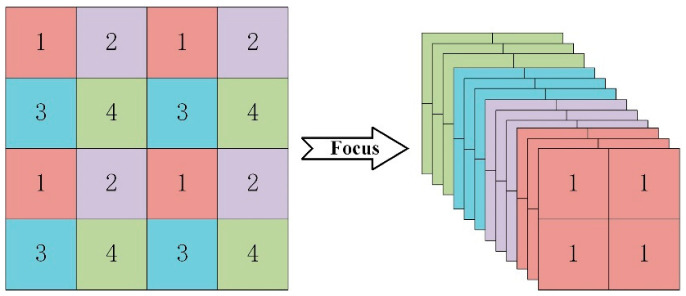
Focus slicing operation.

The backbone part uses the Focus down-sampling, improved BottleneckCSP, and Spatial Pyramid Pooling (SPP) structures to extract feature information of the image. The input RGB image is first split into four parts for two-times down-sampling, then spliced in the channel dimension by the Concat module to obtain a 12-dimensional feature map, which is then sent to the convolution module for feature extraction using a 3 × 3 convolution kernel, obtaining a 32-dimensional feature layer. As shown in Figure 3, Focus down-sampling reduces the size of the feature map by increasing its dimension without sacrificing any information and enhances the network’s performance.

**Figure 4:**
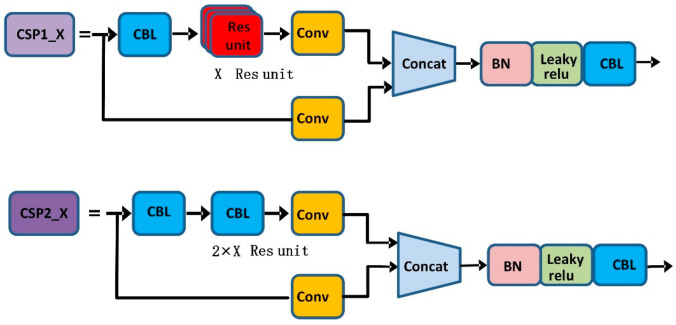
CSP1_X and CSP2_X structures.

**Figure 5:**
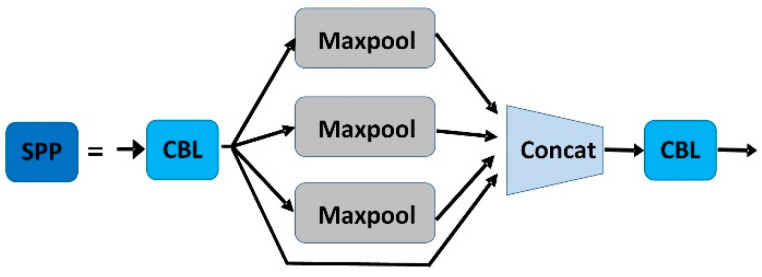
The structure of SPP.

As shown in Figure 4, YOLOv5s contain two types of CSP structures, CSP1_X and CSP2_X. CSP1_X is used in the backbone network, which improves the gradient values of backpropagation between layers and layers, resulting in finer feature granularity. Spatial pyramid pooling can convert feature maps of arbitrary size into feature vectors of fixed size without degrading the recognition accuracy of the images. The structure diagram of SPP is shown in Figure 5.

### 3.3. Neck

**Figure 6:**
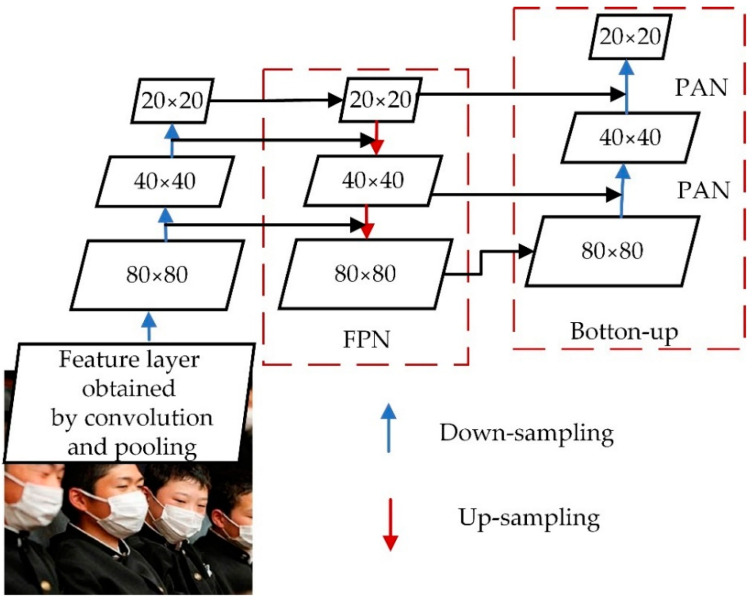
Neck structure diagram.

The CSP2_X structure is used in Neck to improve the network’s feature fusion capability by inserting two residual blocks, which allows the network to reduce computation while maintaining richer feature information. The Neck layer comprises Feature Pyramid Networks (FPN) and Path Aggregation Networks (PAN) [31], as shown in Figure 6. The FPN conveys deep semantic features from top to bottom, whereas the PAN transmits the target’s position information from bottom to top. The model transfers feature information of objects of different sizes through the fusion of top-down and bottom-up feature information, which solves the problem of multiscale object detection.

### 3.4. Head

The loss function and Non-Maximum Suppression form the Head. The loss function of the YOLOv5 model includes three sections: bounding box regression loss, confidence loss, and classification loss. The boundary loss is calculated using the Generalized Intersection over Union (GIOU) function, which considers not only the overlapping area between the actual and predicted boxes but also other non-overlapping areas, allowing it to reflect the size of the distance between the two boxes better than the original IoU and distinguish the difference between the intersection of the two when the IoUs are equal. YOLOv5 uses weighted NMS to filter multiple target anchor boxes and eliminate redundant candidate boxes in the post-processing of target detection.

## 4. Improved YOLOv5 Network Model

This section elaborates on the proposed improved method for YOLOv5-CBD, including attention mechanism, feature extractor, and post-processing methods.

### 4.1. Improved Attention Mechanism

The attention mechanism is a notion presented to mimic the human nervous system. The machine learns to perceive the relevant and unimportant parts of the data, thereby improving the backbone network’s attention to the essential features of the target to be discovered. The attention mechanism assigns varying weights to different feature layers based on the features of the target to be recognized; with the accuracy guaranteed, we prune the model weights and delete redundant information.

Coordinate Attention (CA) captures channel relationships and long-range dependencies with exact location information in two steps: coordinate information embedding and coordinate attention generation. CA divides channel attention into two one-dimensional feature encoding processes that gather features in two spatial directions. Long-range dependencies can be obtained along one spatial direction while exact location information is kept along the other spatial direction. The generated feature maps are then decoded into a pair of direction-aware and position-sensitive attention maps, which can be used in conjunction with the input feature map to enhance the characteristics of the detected object.

**Figure 7:**
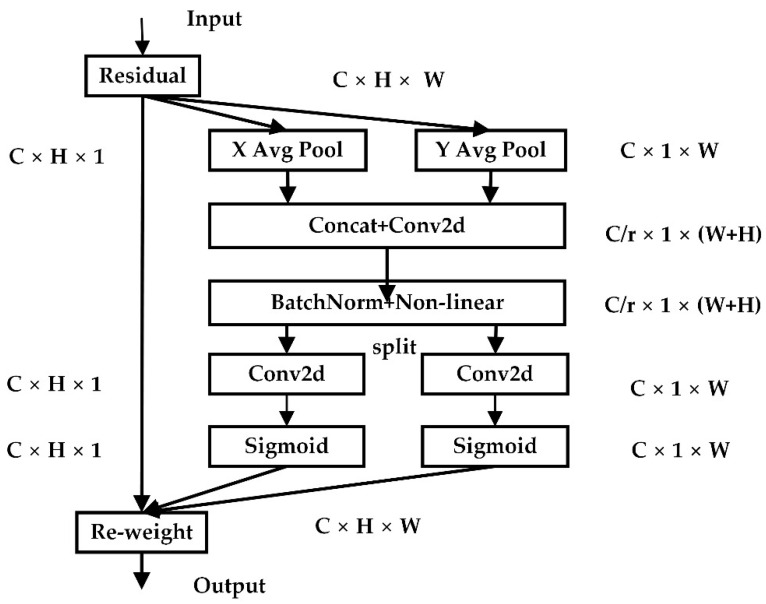
CA module structure.

Figure 7 depicts the CA mechanism implementation process. The input feature maps are pooled globally and averaged from both width and height directions to obtain feature maps in both width and height directions. The calculation formulas are as follows:

(1) $$z_{c}^{h}\left(h\right)=\frac{1}{w}\underset{0\leq i<\omega }{\sum }x_{c}\left(h,i\right)$$

(2) $$z_{c}^{w}\left(w\right)=\frac{1}{H}\underset{0\leq j<H}{\sum }x_{c}\left(j,w\right)$$

The feature maps of the global receptive field’s width and height are spliced together and transmitted to the 1 × 1 convolution module, with the dimension decreased to the original C∕r. After batch normalization, the characteristic figure F1 is fed into the Sigmoid activation function, yielding the characteristic figure f in the form of 1 × (W + H) × C∕r, as shown in Equation (3).

(3) $$f=\delta \left(F_{1}\left(\left[z^{h},z^{w}\right]\right)\right)$$

where $f\epsilon R^{C∕r\times \left(w+h\right)}\;$is the horizontal and vertical intermediate feature map of spatial information, r represents the down-sampling ratio, and δ represents a nonlinear activation function.

The feature map f is convolved by 1 × 1 according to the original height and width to obtain the feature maps $F_{h}$ and $F_{w}$ with the same number of channels as the input x. Using the Sigmoid activation function, obtain the attention weights $g^{h}\;$in the height and $g^{w}$ in the width directions of the feature map. The calculation formulas are as follows:

(4) $$g^{h}=\sigma \left(F_{h}\left(f^{h}\right)\right)$$

(5) $$g^{w}=\sigma \left(F_{w}\left(f^{w}\right)\right)$$

Finally, the original feature map is weighted by multiplication, and the feature map with attention weight in the width and height directions is obtained, as shown in Equation (6).

(6) $$y_{c}\left(i,j\right)=x_{c}\left(i,j\right)\times g_{C\;}^{h}\left(i\right)\times g_{C\;}^{w}\left(j\right)$$

**Figure 8:**
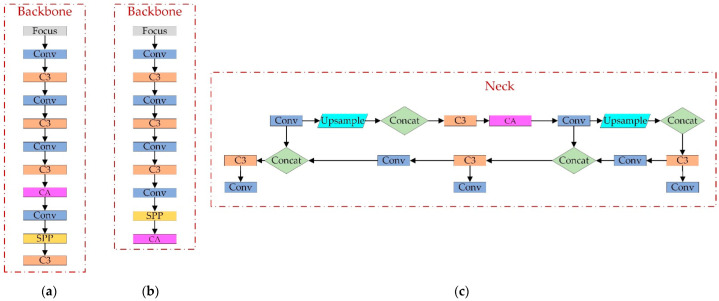
Location of CA additions. (**a**) CA added between C3 and Conv of Backbone; (**b**) CA replaces the last C3 layer of Backbone; (**c**) CA added between C3 and Conv of Neck.

The CA mechanism is a new mechanism that embeds location information into channel attention. At the shallow layer of the network (e.g., image-level), the extracted spatial feature map is too large, and the number of channels is too small, so the obtained channel weights cannot summarize specific features, and the extracted spatial weights are not generalized enough due to the small number of channels. In the later layers of the network, too many channels tend to cause overfitting. More crucially, the closer to the classification layer, the more sensitive the effect of attention is to the classification result, which influences the classification layer decision. Therefore, this paper adds the attention mechanism to the network’s middle part. We conducted ablation experiments to evaluate further the effect of the CA addition position on the algorithm’s performance. The experimental results are shown in Table 1. The positions a–c of CA additions in Table 1 correspond to a–c in Figure 8. Based on the experimental results, we chose Option 3, which has a relatively significant improvement in accuracy.

### 4.2. Improvement of Feature Pyramid Structure

**Figure 9:**
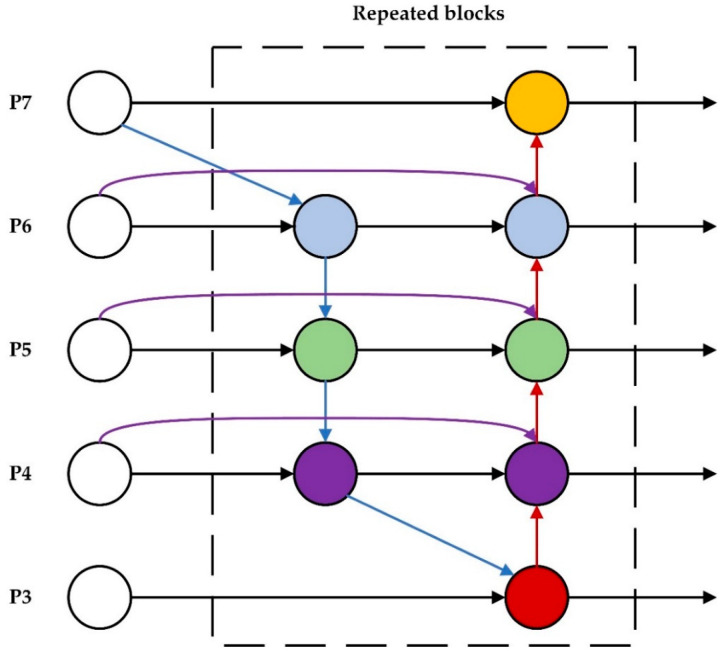
BiFPN implements two optimizations for cross-scale connections.

The Bidirectional Feature Pyramid Network (BiFPN) is a typical complex two-way feature fusion pyramid structure. First, BiFPN streamlines the feature network by deleting intermediary nodes with only one input edge, based on the standard feature pyramid. Second, extra edges are added at the same level between the input and output nodes to fuse more features at a low cost. Finally, BiFPN treats each bidirectional (top-down and bottom-up) path as a single feature network layer and repeats the same layer multiple times to enable higher-level feature fusion. As seen in Figure 9, P3–P7 in the figure represent the fusion features of different levels. Blue arrows indicate the top-down pathway, red arrows indicate the bottom-up pathway, and purple arrows indicate the addition of additional edges to input and output nodes that are on the same level.

Because different input features have different resolutions, different weights should be attributed to the final output at each node where feature fusion is performed. Therefore, BiFPN introduces training weights that add additional weights to each input to adjust the contribution of different inputs to the output feature map. BiFPN chooses weights using Fast normalized fusion, which directly divides the total of all values by the weights and normalizes the normalized weights to the range [0, 1]. With similar optimization results, Fast normalized fusion is up to 30 percent faster than Softmax-based fusion. The calculation formula is as follows:

(7) $$O=\Sigma_{i}\frac{\omega_{i}}{\varepsilon +\Sigma_{j}w_{j}}\cdot I_{i}$$

where$\;I_{i}\;$stands for the characteristics of input, ε is a smaller value to avoid numerical instability, $\omega_{i}\;$≥ 0 is ensured by applying a Relu after each $\omega_{i}$.

This paper uses the BiFPN network as an enhanced feature extraction network to adapt the network’s fusion for different input features and increase the network’s capacity to extract features at different scales.

### 4.3. Improvement in Non-Maximum Suppression

The NMS algorithm sorts the prediction boxes of a given category by confidence level, setting the box with the highest score as the base box. Then, the IoU is calculated with the rest of the boxes, deleting those larger than the set threshold and keeping those smaller than the threshold, and cycling them to eliminate redundant duplicate windows and find the best position. However, in the case of dense targets, the overlapping region of the detection frame is considerable due to mutual occlusion, and the occlusion often leads to incorrect suppression, resulting in target misses.

We integrated DIoU with NMS to improve the missed detection situation. DIoU-NMS considers the IOU of the prediction box and the real box and the distance between the center point of the prediction boundary box and the real boundary box, allowing it to return to the prediction box more accurately. $S_{i}$ and $R_{DIOU}$ are calculated as:

(8) $$s_{i}=\{\begin{array}{c} s_{i},\;IOU-R_{DIOU}\left(M,B_{i}\right)<\varepsilon \\ 0,IOU-R_{DIOU}\left(M,B_{i}\right)\geq \varepsilon \end{array}$$

(9) $$R_{DIOU}=\frac{\rho^{2}\left(b,b^{g_{t}}\right)}{c^{2}}$$

where $s_{i}$ is the classification score, $R_{DIOU}$ is the penalty factor of the DIoU-NMS algorithm, ε represents the threshold of NMS, $\rho \left(b,b^{g_{t}}\right)$ represents the Euclidean distance between the predictor box with the highest confidence level and the center coordinate of the predictor box, and c denotes the diagonal length of the smallest enclosing box that contains both predictor boxes.

### 4.4. YOLOv5-CBD Network Structure

**Figure 10:**
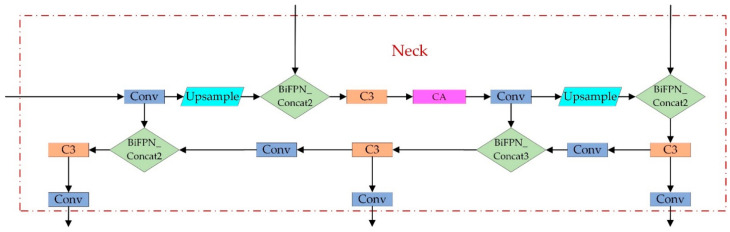
The structure of the improved module.

We obtained the improved YOLOv5 algorithm through the theoretical analysis and research from the previous chapters, and the structure of the improved module is shown in Figure 10.

## 5. Experiments and Results

### 5.1. Dataset

The dataset in this paper consists of the open-source dataset RMFD [32], AFLW, and a web crawler. The dataset includes densely populated areas such as streets and subways, enhancing the number of small-scale and dense targets. The final data set contains 3010 images after screening, cleaning, and other procedures, including 2410 images in the training set and 600 in the validation set. The dataset is annotated in the YOLO format using the tag program LabelImg, with three labeling categories: face, mask, and mask incorrectly. The sample distribution of different categories in the dataset is shown in Table 2, where objects represent the number of instances.

**Figure 11:**
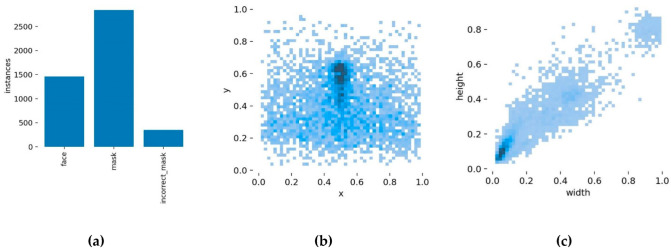
The visualization results of the analysis of the dataset. (**a**) The distribution of object categories in the dataset; (**b**) the distribution of object centroid locations; (**c**) the distribution of object sizes.

Figure 11 depicts the visualization results of the dataset analysis, where (a) represents the distribution of object categories in the dataset, (b) represents the distribution of object centroid locations, with the horizontal and vertical coordinates indicating the location of the centroids, and (c) represents the distribution of object sizes, with the horizontal and vertical coordinates indicating the width and height of the objects.

### 5.2. Evaluation Metrics

In our experiments, precision, recall, mean average precision (*mAP*), and processing time are employed as evaluation metrics to investigate the performance of each network. Before introducing these metrics, the following concepts are introduced: True Positives (TP) refer to the number of positive samples correctly assigned by the classifier; True Negatives (TN) refer to the number of negative samples correctly assigned; False Positives (FP) refer to the number of positive samples misclassified; False Negatives (FN) refer to the number of negative samples misclassified. Intersection over Union (IOU) measures the degree of overlap between the candidate bound and the ground truth bound.

**Figure 12:**
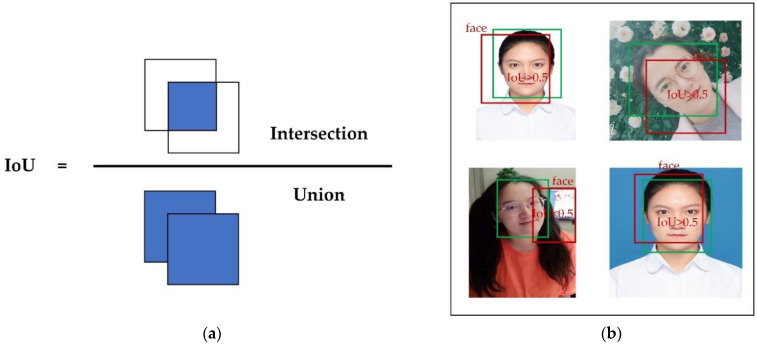
IoU calculation and applications. (**a**) IoU calculation example diagram; (**b**) dividing samples according to IoU thresholds.

Take Figure 12b as an example. The green box in the figure indicates the real target box and the red box indicates the predicted box for recognition. We set the threshold value of IoU as 0.5; when the IoU value is greater than 0.5, the prediction result is correct, otherwise, the prediction result is wrong. Based on the concepts of TP, FP, and FN, we know that TP = 3, FP = 1, and FN = 1 in Figure 12b. Substituting these numbers into Equations (10) and (11), we can calculate the precision and recall.

Precision is the proportion of correct positive samples in the anticipated data set to the number of positive samples predicted by the model. The recall is the proportion of right positive samples in the predicted data set to the actual number of positive samples in the predicted data set. The calculation formulas of precision and recall are given in Equations (10) and (11), respectively.

(10) $$Precision=\frac{TP}{TP+FP}$$

(11) Recall=TPTP+FN

Average Precision (*AP*): the region of the graph is bounded by the P-R curve and the coordinate axis. The calculation formula is as follows:

(12) $$AP=\overset{n-1}{\underset{i=1}{\sum }}\left(r_{i+1}-r_{i}\right)p\left(r_{i+1}\right)$$

Mean Average Precision (*mAP*): the mean of all categories in the dataset’s average precision. The calculation formula is as follows:

(13) $$mAP=\frac{1}{N}\overset{N}{\underset{i=1}{\sum }}AP_{i}$$

In this experiment, *N* = 3 represents the number of target detection categories.

### 5.3. Training

#### 5.3.1. Experimental Environment and Parameter Setting

The parameters for each model are identical, with the number of network iterations set to 100 and the iteration batch size set to 4. After each iteration, we save the model weights, the ideal model weights are finally obtained, and the model weights are loaded in the subsequent detection process. The Warmup is used to preheat the learning rate during model training, eliminating oscillation and maintaining deep model stability by reducing the overfitting of small-batch data in the early phases of the model. After preheating, the learning rate is adjusted using the cosine annealing process. The training platform parameters are detailed in Table 3.

#### 5.3.2. Training Result

**Figure 13:**
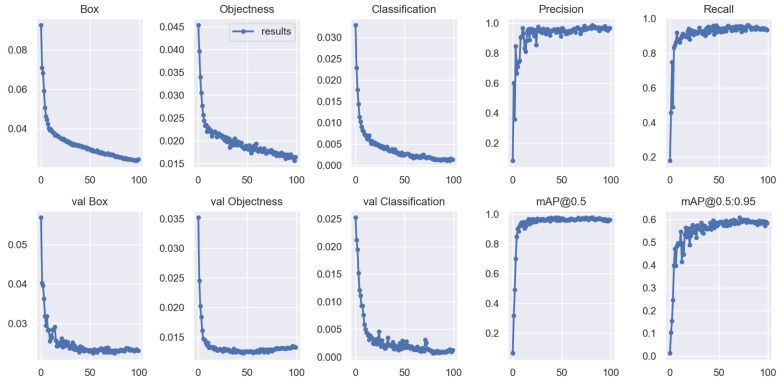
Plots of Box loss, Objectness loss, Classification loss, Precision, Recall and mean Average Precision (*mAP*) over the training epochs for the training and validation set.

Training the model for 100 epochs, we can obtain the training results of the improved model, and the different performance metrics of the training and validation sets are shown in Figure 13.

After about 50 epochs, the model reached a plateau regarding precision, recall, and mean average precision. The Box, Objectness, and Classification losses decreased drastically in the first 50 epochs of network training before stabilizing at around 80. Therefore, we choose the best weight obtained after training 100 epochs as the weight for mask-wearing detection.

### 5.4. Analysis of Experimental Results

#### 5.4.1. Comparison Experiment

The YOLOv5-CBD model was compared with Fast R-CNN, SSD, YOLOv3, YOLOv4, and the original YOLOv5 algorithm with default parameters to verify the accuracy and reliability of the proposed method. Considering the requirements of practical scenarios, we choose three metrics—mAP@0.5, recall, and detection speed—as the measurement criteria. These models are trained and validated on the same dataset, and the experimental results are shown in Table 4.

As shown in Table 4, the YOLOv5s model in the detection algorithm has higher detection accuracy and recall than the two-stage Fast-RCNN and the one-stage SSD, YOLOv3 and YOLOv4. Compared to Fast R-CNN, SSD, YOLOv3, and YOLOv4, the YOLOv5s model increased by 11.7%, 16.3%, 3.4%, and 2.5% in mAP@0.5 and 9.8%, 14.1%, 5% and 1.1% in recall rate. The one-stage detection algorithm YOLOv5s is the quickest, with an FPS of 31.3, much greater than the two-stage detection algorithm and the other one-stage algorithms in the table. Our improved YOLOv5-CBD model improves mAP@0.5 and recall by 2.1% and 3.8%, respectively, over YOLOv5s. The FPS of YOLOv5-CBD reaches 29, 2.3 lower than the YOLOv5s model, but still meets the real-time requirement. YOLOv5-CDB outperforms the other target detection algorithms in the table when the metrics of mAP@0.5, recall, and inference time are considered together.

#### 5.4.2. Ablation Experiment

We conducted ablation experiments in this study to comprehensively validate the optimization effect of each improvement module and to further assess the effects of the improvement technique on the YOLOv5 algorithm. Table 5 shows the results of the experiment.

Experiment 1 is the original YOLOv5, with a detection precision of 94.7%, recall of 91.4%, mAP@0.5 of 94.6%, and FPS of 31.3. Experiment 2 added the CA mechanism to the neck layer, which boosted the mAP@0.5 by 0.6% and enhanced detection precision and recall by 1.1% and 1.4%, respectively. In contrast, the detection speed was reduced by 1.2 FPS. Experiment 3 shows an increase in detection accuracy and recall and a 1.5% rise in mAP@0.5 over baseline, suggesting that the improved feature fusion network can improve model performance. Experiment 4 integrated DIoU with NMS in the post-processing stage, resulting in a 5.4% improvement in FPS above YOLOv5, significantly improving model inference efficiency. Experiment 5 demonstrates the effectiveness and superiority of YOLOv5-CBD by achieving 96.7% mAP@0.5, a 2.1% improvement over the original YOLOv5 network.

### 5.5. Analysis of Detection Results

**Figure 14:**
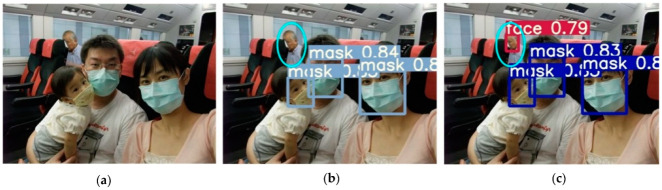
Detection of obscured targets. (**a**) Initial image sample; (**b**) YOLOv5 detect results; (**c**) YOLOv5-CBD detect results.

**Figure 15:**
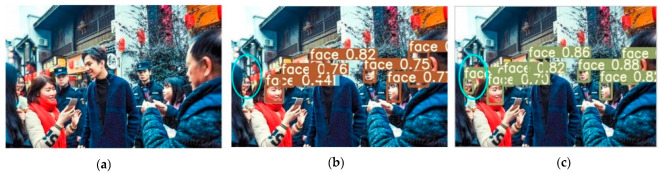
Detection of cross-dense targets. (**a**) Initial image sample; (**b**) YOLOv5 detect results; (**c**) YOLOv5-CBD detect results.

**Figure 16:**
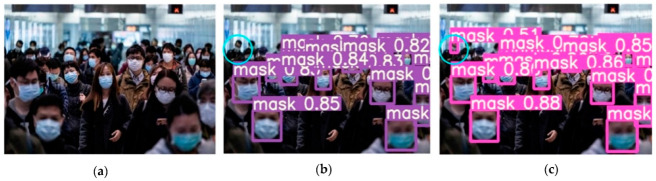
Detection of small targets at long distances. (**a**) Initial image sample; (**b**) YOLOv5 detect results; (**c**) YOLOv5-CBD detect results.

**Figure 17:**
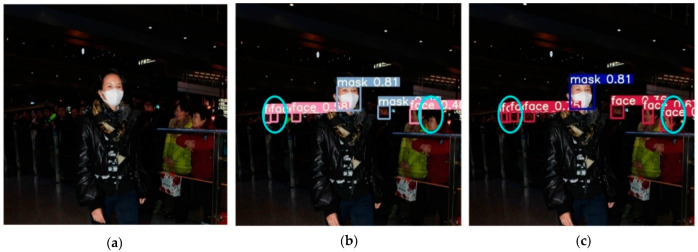
Detection of low light scenes. (**a**) Initial image sample; (**b**) YOLOv5 detect results; (**c**) YOLOv5-CBD detect results.

Several images from the dataset were chosen for testing to verify the model’s viability, and the accompanying figure shows the detection results of the YOLOv5-CBD and YOLOv5 algorithms in different scenarios. Figure 14 shows the recognition of the occluded target, where the face occluded by the seat is not detected in Figure 14b, but the occluded target is detected in Figure 14c. Figure 15 shows the detection of cross-dense targets with overlapping face targets is missed in Figure 15b and correctly detected with higher accuracy in Figure 15c. Figure 16 shows a station with dense targets, and it can be observed from Figure 16b,c that the improved algorithm can better detect and identify small-scale targets for long-range. Figure 17 shows the target detection in a low-light scene, where Figure 17b misses the faces in dark areas and Figure 17c correctly detects more targets.

To summarize, the YOLOv5-CBD model outperforms the YOLOv5 model in detecting small-scale and dense targets and having strong robustness, resulting in superior performance and more precise detection and recognition.

## 6. Conclusions

This paper proposes an improved mask-wearing recognition algorithm based on YOLOv5. First, we added an attention module to the neck layer, allowing the network to extract critical feature information in complex scenes quickly. Then, we introduced BiFPN to enhance the adjustment ability of the detector for objects of different scales by fusing features of different scales. Finally, we combined DIoU with NMS to reduce missed detection of overlapping targets. The mAP@0.5 of the YOLOv5-CBD model is 96.7%, a 2.1% improvement over the YOLOv5 model, and the FPS is 29, indicating that the algorithm reaches excellent accuracy and has the potential for real-time detection.

In future work, we will add more heavily obscured and half-face images to the dataset to extract more features and train the network’s feature extraction capacity. Furthermore, we plan to replace the Backbone network with MobileNetV3 [33] to create a lightweight network with higher performance to match the lightweight requirements for target detection in mobile or embedded devices.

## Figures and Tables

**Table 1:** Ablation experiment of Coordinate Attention.

NO	Location of CA	Precision	Recall	mAP%@0.5
0	Not added	94.7%	91.4%	94.6%
1	Backbone (a)	94.4%	90.6%	93.9%
2	Backbone (b)	94.6%	91.4%	94.5%
3	Neck (c)	95.8%	92.8%	95.2%

**Table 2:** Distribution of different types of samples in the data set.

Dataset	Face	Mask	Mask Incorrectly
Training Set Objects	1464	2841	353
Validation Set Objects	329	591	152
Total	1793	3432	505

**Table 3:** Hardware and software platforms.

Hardware and Software Platforms	
The operating system	windows10
CPU	Intel(R) Xeon(R) CPU E5-2699 v4
GPU	NVIDIA Quadro M5000
Development platform	PyTorch1.11.0
Development of language	Python3.8

**Table 4:** Performance comparison results of different algorithms.

Model	Precision	Recall	mAP@0.5	One Image Test Time/s	FPS
Fast R-CNN	84.1%	81.6%	82.9%	0.244	4.1
SSD	80.1%	77.3%	78.3%	0.12	8.3
YOLOv3	93.1%	86.4%	91.2%	0.075	13.3
YOLOv4	93.6%	90.3%	92.1%	0.052	19.2
YOLOv5s	94.7%	91.4%	94.6%	0.032	31.3
YOLOv5-CDB	96.3%	95.2%	96.7%	0.034	29

**Table 5:** Results of ablation experiments.

NO	Model	Precision	Recall	mAP@0.5	Inference Time/s	FPS
1	YOLOv5s	94.7%	91.4%	94.6%	0.032	31.3
2	YOLOv5 + CA	95.8%	92.8%	95.2%	0.033	30.1
3	YOLOv5 + BiFPN	96.5%	93.3%	96.1%	0.033	30.1
4	YOLOv5 + DIOU-NMS	96.3%	92.8%	95%	0.030	33
5	YOLOv5-CBD	96.3%	95.2%	96.7%	0.034	29

## Data Availability

Not applicable.
